# Supplementary material for: Transcribed Ultraconserved Regions Are Associated with Clinicopathological Features in Breast Cancer
Source: Biomolecules. 2022 Jan 26;12(2):214. doi: 10.3390/biom12020214 (PMC8961524; doi:10.3390/biom12020214)
Supplement: Supplementary file 1 [file biomolecules-12-00214-s001.zip › biomolecules-1527973 supp/Supplementary figures.pdf]

Supplementary Figures

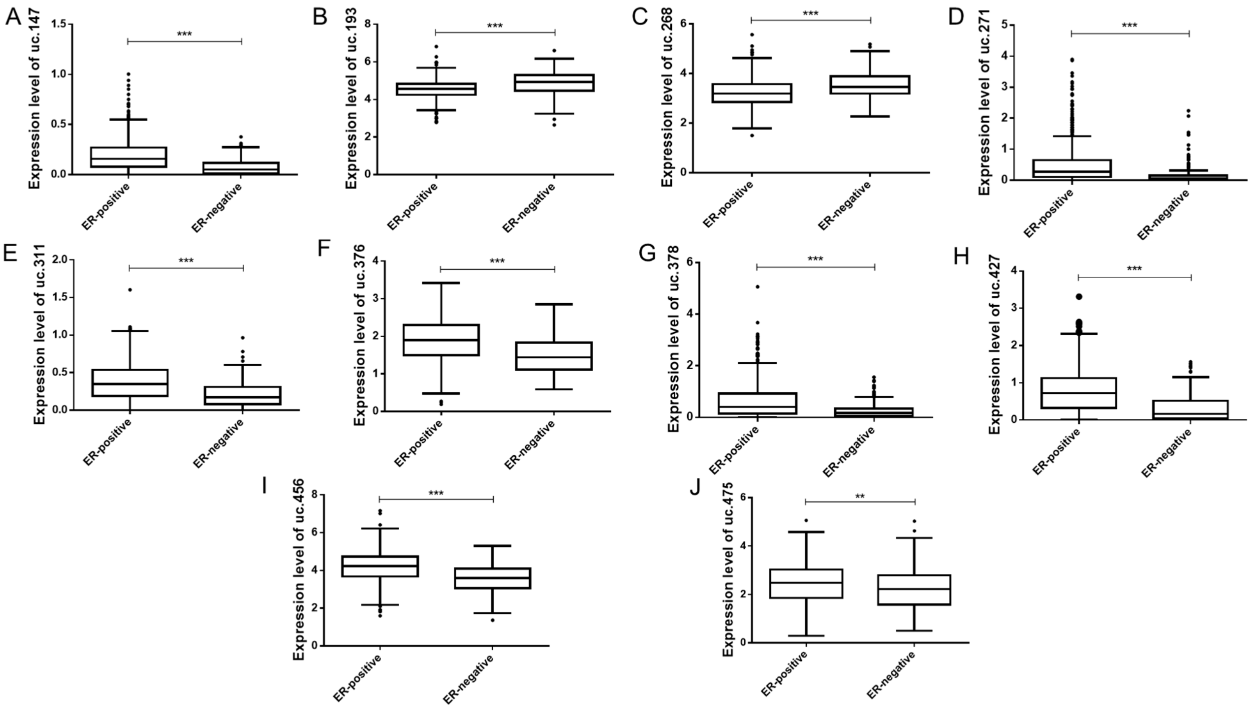

Figure S1. Comparison of T-UCRs expression to estrogen receptor from TCGA data. (**A**) Differential expression level of uc.147 (B) uc.193, (C) uc.268, (D) uc.271, (E) uc.311, (F) uc.376, (G) uc.378, (H) uc.427, (I) uc.456 and (J) uc.476 in ER-positive versus ER-negative. *$p < 0.05$, **$p < 0.01$, *** $p < 0.001$, **** $p < 0.0001$.

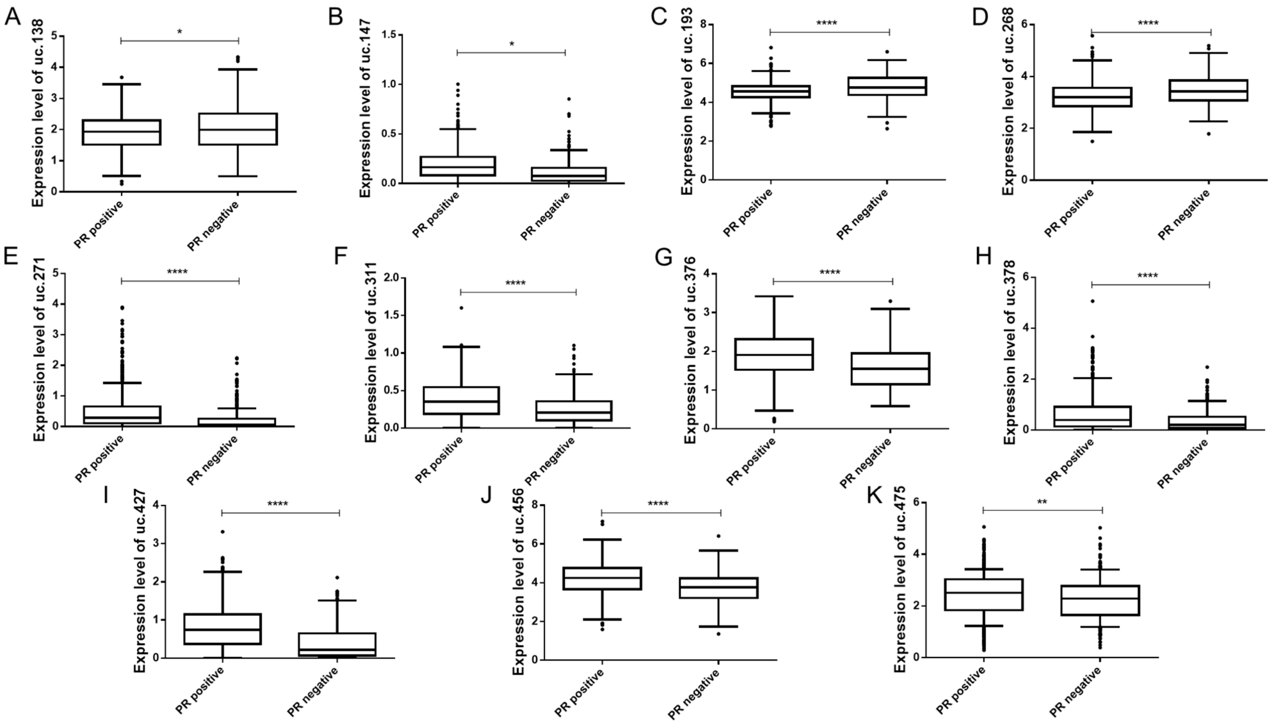

Figure S2. Comparison of T-UCRs expression to progesteron receptor from TCGA data. Differential expression level of (**A**) uc.138, (B) uc.147, (C) uc.193, (D) uc.268, (E) uc.271, (F) uc.311, (G) uc.376, (H) uc.378, (I) uc.427, (J) uc.456 and (K) uc.475 in PR-positive versus PR-negative. *$p < 0.05$, ** $p < 0.01$, *** $p < 0.001$, **** $p < 0.0001$.

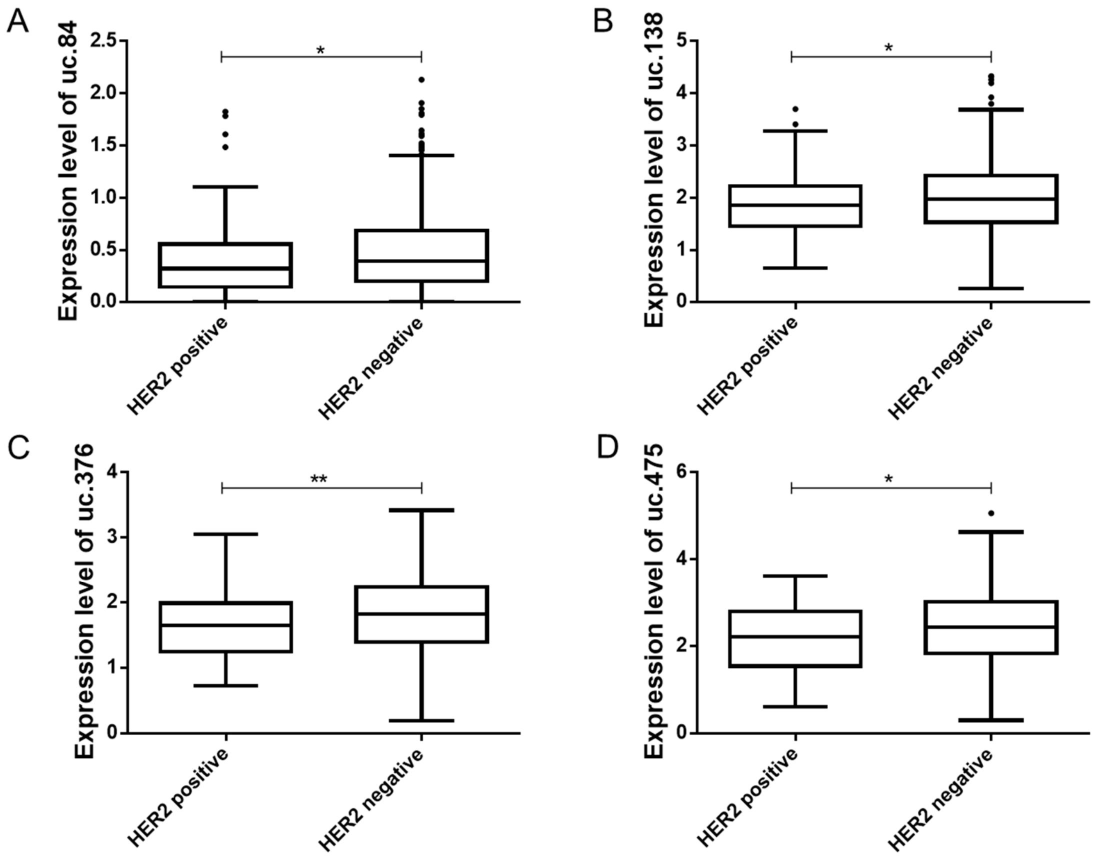

Figure S3. Comparison of T-UCRs expression to progesteron receptor from TCGA data. Differential expression level of (A) uc.84, (B) uc.138, (C) uc.376, (D) uc.475 in HER2-positive versus HER2-negative. *$p < 0.05$, ** $p < 0.01$, *** p < 0.001, **** $p < 0.0001$.
